# Necdin Enhances Myoblasts Survival by Facilitating the Degradation of the Mediator of Apoptosis CCAR1/CARP1

**DOI:** 10.1371/journal.pone.0043335

**Published:** 2012-08-14

**Authors:** Stephanie François, Cristina D'Orlando, Tiziana Fatone, Thierry Touvier, Patrizia Pessina, Raffaella Meneveri, Silvia Brunelli

**Affiliations:** 1 Department of Experimental Medicine, University of Milano-Bicocca, Monza, Italy; 2 E. Medea Scientific Institute, Bosisio Parini, Lecco, Italy; 3 Division of Regenerative Medicine, Stem Cells and Gene Therapy, San Raffaele Scientific Institute, Milan, Italy; University of Rome La Sapienza, Italy

## Abstract

Regeneration of muscle fibers, lost during pathological muscle degeneration or after injuries, is sustained by the production of new myofibers by means of the satellite cells. Survival of the satellite cells is a critical requirement for efficient muscle reconstitution. Necdin, a member of the MAGE proteins family, is expressed in satellite cell-derived myogenic precursors during perinatal growth and in the adult upon activation during muscle regeneration, where it plays an important role both in myoblast differentiation and survival. We show here that necdin exerts its pro-survival activity by counteracting the action of the pro-apoptotic protein Cell Cycle Apoptosis Regulatory Protein (CCAR1/CARP1) that we have identified as a new molecular interactor of necdin by two-hybrid screening. Necdin is responsible for the maintenance of CCAR1 protein levels, by implementing its ubiquitination and degradation through the proteasome. Taken together, these data shed new light on the molecular mechanism of necdin anti-apoptotic activity in myogenesis.

## Introduction

Skeletal muscle tissue is characterized by a very slow turnover that accelerates however upon certain physiological stimuli or in pathological conditions, such as primary myopathies, leading to an extensive repair process aimed at preventing the loss of muscle mass. The initial phase of muscle repair is characterized by necrosis of the damaged tissue and activation of an inflammatory response [1]. Immediately after, local cues, produced by growth factors and inflammatory cytokines released by infiltrating cells, lead to the activation of quiescent myogenic cells, the satellite cells, located beneath the basal lamina of muscle fibers, that start to proliferate, differentiate and fuse, leading to new myofiber formation and reconstitution of a functional contractile apparatus. A delicate balance between cell proliferation, differentiation and fusion is required for the correct muscle regeneration to occur. Survival of the satellite cells is indeed a critical requirement for efficient muscle reconstitution. Disruption of the muscle sarcolemma leads to generation of several pro-apoptotic cues, including the release of cytokines and oxidants species by the infiltrating neutrophils and activated macrophages, that can promote additional muscle damage by stimulating apoptosis of myogenic stem cells [2], [3]. Several molecules have been found to show a pro-apoptotic or anti-apoptotic effect on myogenic stem cells in physiological or pathological conditions, even if only limited information exists about the actual molecular mechanisms and signalling molecules that regulate the decision to self-renew, to differentiate or to die. Pax3 and Pax7, two critical master regulator genes, appear to have roles on both the specification and survival of myogenic precursors, having distinct and overlapping functions, and cells failing to express Pax3 or Pax7 die or assume a non-myogenic fate. In particular Pax3 pro-survival function is mostly required during embryogenesis. Pax3 regulates neural tube closure by inhibiting p53-dependent apoptosis [4] and Pax3 mutant somitic cells undergo extensive apoptosis [5]. In the adult as well, it has been recently shown that Pax3 upregulation in MyoD^−/−^ myoblasts, together with Bcl2 and Bclx, are responsible for the resistance to apoptosis [6]. Pax7 show an anti-apoptotic effect in postnatal life. The numbers of satellite cells fall during postnatal development in Pax7 mutant mice [7], [8] and expression in myoblasts of a dominant-negative form of Pax7, but not of Pax3, leads to many cells dying [9]. Efficient self-renewal of the satellite cell pool after muscle injury is also critical for the maintenance of the tissue homeostasis. Sprouty1 (Spry1), a receptor tyrosine kinase signalling inhibitor, is required to maintain the quiescent stem cell pool during muscle repair [10]. It is expressed in quiescent satellite cells, downregulated in proliferating myogenic cells after injury, and re-induced as cells re-enter quiescence. In absence of Spry1 function, regenerating muscles show increased number of apoptotic myogenic cells [10]. Other pleiotropic signals, such as the Ang1/Tie2 system, also promote satellite cell survival, and contribute to the regulation of stem cell quiescence and self-renewal in skeletal muscle [11].

We have identified necdin as a critical key player in muscle regeneration [12]. Necdin is a member of the MAGE family [13], a large family of proteins initially isolated from melanomas, characterized by a large central region termed the MAGE homology domain MHD. We have shown that necdin is expressed in satellite cells and is able to sustain efficient muscle differentiation and regeneration by acting on two different pathways: on myoblast differentiation, by direct transcriptional regulation of myogenin and by protecting myoblasts from cell death. In necdin loss of function models, regenerating muscles show an increased level of myoblasts cell death, and myoblasts are more sensitive to apoptotic stimuli *in vitro*, showing activation of caspases 9 and 3 [12]. More recently we have shown that necdin is able to accelerate and enhance myogenic differentiation of the vessel-associated stem cells mesoangioblasts [14], [15] and to promote their survival *in vitro* and in transplantation assays, thus leading to a more efficient reconstitution of the dystrophic muscle [16]. Furthermore in models of muscle atrophy, we found that necdin carries out a protective effect on muscle mass loss mainly via interference with TNF-α signalling at various levels, including regulation of expression of TNFR1 and p53, and regulation of the activity of caspase 3 and caspase 9 [17].

Whether necdin acts directly or indirectly, transcriptionally and/or post-transcriptionally and which are the molecular mechanisms involved, is still largely unknown, as is the role of the other members of the MAGE family. Only very recently several MAGE proteins were identified to interact with RING domain proteins, including a sub-family of E3 ubiquitin ligases, suggesting that MAGE proteins might function in the ubiquitination cascade mediated by their RING interacting partners [18].

By using a Two-Hybrid assay in yeast we were able to identify a new molecule able to bind necdin in myogenic cells. We show that necdin interacts with Cell Cycle Associated Regulating Protein 1 (CCAR1/CARP1) in muscle progenitor cells and it is able to mediate its degradation by the proteasome therefore counteracting CCAR1 pro-apoptotic action.

## Results

### CCAR1/CARP1 is a novel molecular binding partner of necdin

To identify new protein binding partners that interact with necdin in myogenic cells, we performed a two-hybrid screening in yeast. We generated a cDNA library, containing LD recombination sequences from RNA of proliferating primary myoblasts isolated from newborn mice (C57Bl6) (LD-library Clontech). Necdin ORF was cloned in frame with the Gal4 DNA binding domain in the pGBKT7 vector, and the plasmid was co-transformed together with the cDNA library and with the linearized pGAD-Rec plasmid, containing the Gal4 activation domain, in AH109 cells using the Clontech system. Transformants were grown first on leucine-tryptophan and histidine deficient synthetic dropout (SD) medium plates. The 2mm colonies were then grown on leucine, histidine, tryptophan and adenine deficient dropout medium supplemented with X-Gal to test the β-galactosidase activity. 600 resistant clones and X-Gal positive colonies were analysed by colony-PCR using the LD primers. 225 PCR fragments longer than 500bp were subsequently sequenced. The sequence we found to be more frequent (6 times) corresponded to Cell Cycle and Apoptosis Regulatory protein-1, or CCAR1/CARP1 or CCAR1 (MGI:1914750). CCAR1 is a perinuclear protein that mediates apoptosis signalling by different agents, leading to the activation of caspase 9 and 3 [19], [20], [21]. The cloned portion of CCAR1 that was found to interact with necdin corresponds to amino-acids 637–822.

**Figure 1:**
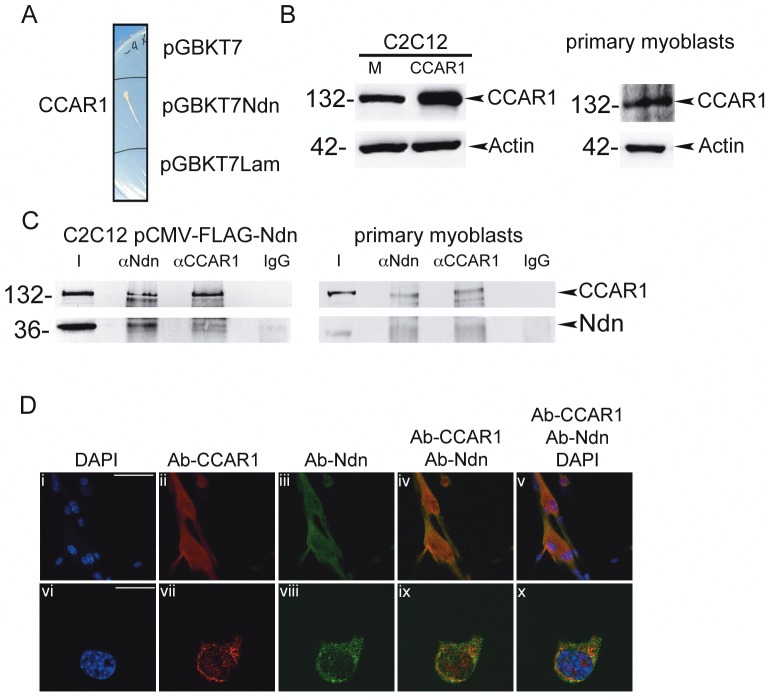
Necdin interacts with CCAR1/CARP1. A) Necdin interacts with CCAR1 in yeast in mating experiments. The plasmid pGBKT7-containing either the cDNA sequence for necdin, laminin (as unrelated sequence) or the empty vector (as negative control) were transformed in Y187 mat-α while AH109 mat-a cells were transformed with the plasmid pGAD-Rec CCAR1 (previously extracted and sequenced from the specific clone of two hybrid in yeast result). After mating only the diploids cells were able to grow on selective medium (SD/-Leu, -His,-Trp, -Ade). B) Representative western blot using antibodies specific for CCAR1 or β-actin as control, in mock-transfected C2C12 (M), C2C12 transfected with pSG5-HA-CCAR1 vector (CCAR1) and in primary myoblasts isolated from C57Bl6 newborn mice. C) Interaction of necdin and CCAR1 in C2C12 myoblasts and primary myoblast cells. Co-IP experiments were performed on protein extract from C2C12 transfected with pCMV-FLAG-Necdin (C2C12 pCMV-FLAG-Ndn) or primary myoblasts from C57/Bl6 newborn mice. Proteins were immunoprecipitated using the polyclonal anti-Ndn (α-Ndn) or polyclonal anti-CCAR1 (α-CCAR1) and with a non-specific rabbit antisera as control (IgG). Necdin and CCAR1 were detected in immunoprecipitated samples using specific antibody. Input (I) represents 10% of the immunoprecipitated proteins. D) Co-localization of necdin and CCAR1 in primary myoblasts of wt (C57/Bl6) mice. Images taken at confocal laser scanning miscroscope at 40x (i–v) and 63x (zoomed 1.67 times) magnification. Nuclei are stained with DAPI (i, vi; blue). Panels shows immunostaining experiment using the polyclonal anti-CCAR1 (ii, vii, Ab-CCAR1-red) or monoclonal anti-Ndn (iii, viii; Ab-Ndn-green). Panel iv shows the merged images of ii and iii and panel ix the merged images of vii and viii (Ab-CCAR1 + Ab-Ndn-yellow). Panel v shows the merged images of i, ii and iii and panel x the merged images of vi, vii, viii, (Ab-CCAR + Ab-Ndn + DAPI). Scale bar (i–iv)  = 17 µm; (vi–x)  = 41,75 µm.

To further validate the interaction in yeast we first isolated the pGAD-Rec CCAR1 plasmid from the specific AH109 cell clone. The plasmid was purified, re-sequenced to confirm its identity, and re-transformed in AH109 mat-a cells, while pGBKT7-Necdin was transformed in Y187 mat-α cells. After mating, the diploids were able to grow in selective medium, thus confirming the interaction (Fig. 1A).

### CCAR1 is expressed in primary myoblasts and C2C12 myoblast cell line

Several cell lines and tissue have been reported to express CCAR1. Western blot analysis show that CCAR1 is expressed in C2C12 cells and in primary myoblasts isolated from muscle of 2 weeks old C57/Bl6 mice (Fig. 1B).

To confirm that CCAR1 and necdin interact in these cells we decided to perform a co-immunoprecipitation (co-IP) analysis. Co-IP analysis was performed on protein extracts from both C2C12 and primary myoblasts.

To validate this interaction in the C2C12 myoblast cell line, where necdin is poorly expressed, C2C12 cells were transfected with pCMV-FLAG-Necdin. Indeed, we were able to detect the presence of necdin-FLAG in sample where we immunoprecipitated CCAR1 using a specific polyclonal antibody anti-CCAR1. Conversely, when we immunoprecipitated necdin with its specific polyclonal antibody, we identified CCAR1 in the immunoprecipitated sample by western blot (Fig. 1C, left panel).

In primary myoblasts we immunoprecipitated necdin using a polyclonal anti-Ndn, and CCAR1 was detected in the immunoprecipitated sample using a CCAR1 specific polyclonal antibody; also in these cells we were able to detect the presence of necdin- in sample where we immunoprecipitated CCAR1 using the specific polyclonal antibody anti-CCAR1. (Fig. 1C, right panel). These data show that indeed in myoblast cells, both primary cells and cell line, necdin and CCAR1 belong to the same protein complex.

In addition, by immunofluorescence on proliferating primary myoblasts using specific antibodies, we could observe that necdin and CCAR1 display overlapping cellular localization in the cytoplasm and around the nucleus (Fig. 1D).

### Necdin protects myoblasts from apoptosis by counteracting CCAR1/CARP1 action

CCAR1 protein has been shown to be a target of apoptosis signalling, and in turn, it promotes apoptosis by activating caspase-9 [20], [21], [22].

To get insight into the function of the necdin-CCAR1 interaction and to verify if this mediates necdin anti-apoptotic action we decided to study how the expression of the two proteins influences cells death.

**Figure 2:**
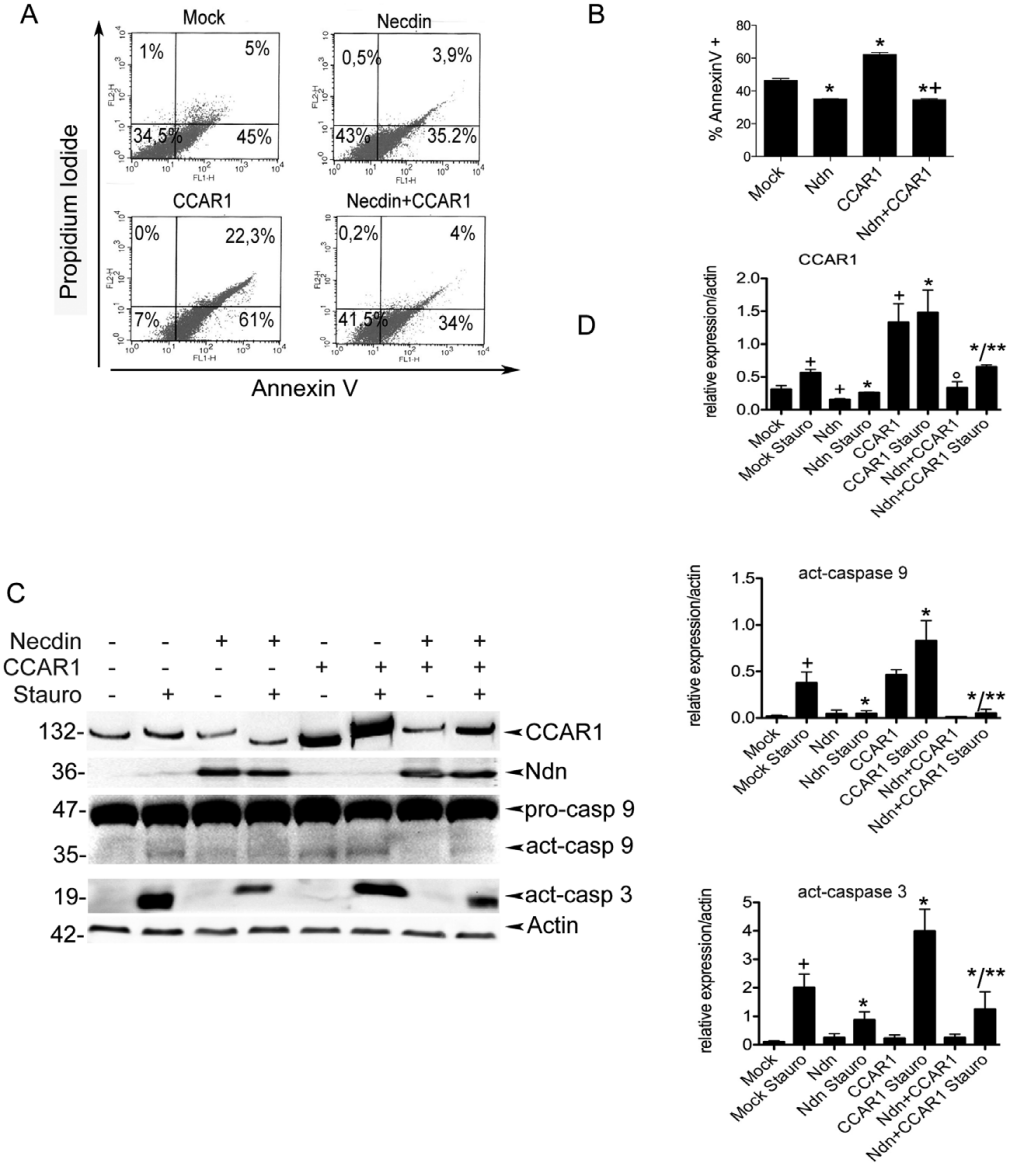
Necdin protects myoblasts from apoptosis by inhibiting CCAR1 activity. A) C2C12 myoblasts were transfected with pCMV-FLAG (Mock) pCMV-FLAG-Necdin (Ndn), and /or pSG5-HA-CCAR1 (CCAR1, Ndn + CCAR1). After 60 h all cells were treated with 200 nM Stausporine for 12 h and cells were collected to measure Annexin V-FITC and non vital-dye propidium iodide (PI) by flow cytometry. Dot plot analyses are representative of four similar experiments. Percentage indicates the proportion of cells that are PI and Annexin V double negative (bottom left section of the plot), PI and Annexin V double positive (top right), PI positive (top left), and Annexin V positive (bottom right). B) The graph on the right show percentage of cells positive for AnnexinV (indicating cells in early apoptosis). Data are representative of four independent experiments (*p<0.02 respect to mock transfected cells; + *p<*0.01 respect to CCAR1 transfected). C) Representative western blot showing expression of pro-caspase 9 (pro-casp 9), activated caspase 9 (act-casp 9), activated caspase 3 (act-casp 3), CCAR1, necdin (Ndn) and β-actin as loading control, on C2C12 myoblasts transfected with pCMV-FLAG-Necdin, pCMV-FLAG and/or pSG5-HA-CCAR1, treated for 3 h with 200 nM Staurosporine. D) Graphs show mean values ± s.e.m. obtained from the ratio of densitometric values of protein/β-actin bands on the blots in (B). Data are representative of three independent experiments. (+ *p*<0.02 vs mock transfected ; * *p*<0,01 vs mock transfected Staurosporine treated; °*p*<0,05 vs CCAR1 transfected; ** vs CCAR1 transfected Staurosporine treated).

To this end C2C12 myoblasts were first transfected with pCMV-FLAG (Mock), pCMV-FLAG-Necdin (Ndn), pSG5-HA-CCAR1 (CCAR1) or both pCMV-FLAG-Necdin and pSG5-HA-CCAR1 (N+C). After 60 h cells were exposed for 3 h or 12 h to the cytotoxic molecule Staurosporine (200 nM). Cell death was determined by measuring both Annexin V levels of phosphatidylserine exposed on the outer leaflet of the plasma membrane and propidium iodide incorporation after 12 h treatment. Results obtained are summarized in representative dot plot analyses and their relative quantification, shown in Fig. 2A, B. As previously reported [12], [17]. Staurosporine mediated cell death is decreased when necdin is overexpressed, while exposure to Staurosporine increased cell death in CCAR1 overexpressing C2C12 respect to mock transfected cells. This effect is counteracted by co-expressing necdin.

This result was also confirmed by the analysis of caspases activation by western blot, after 3 h of Staurosporine treatment. Staurosporine treated C2C12 cells transfected only with pSG5-HA-CCAR1 showed a high level of activation of caspases 3 and 9 (Fig. 2C, D), while the apoptotic pathway is less activated when necdin is overexpressed, as already reported [12], [17]. Again in cells transfected with both necdin and CCAR1, levels of activation of caspases are reduced to the levels of mock-transfected cells, indicating that necdin counteracts CCAR1 pro-apoptotic action. Interestingly, exposure to Staurosporine lead to an increased level of endogenous CCAR1 and this effect is counteracted by necdin co-expression (Fig. 2C, D).

These results suggest that necdin can inhibit the pro-apoptotic action of CCAR1.

Furthermore we observed that CCAR1 levels are decreased when necdin is overexpressed (Fig. 2C, D).

### Necdin leads to a decreased level of CCAR1 protein by mediating its proteasomal degradation

To further get insight into the mechanisms by which necdin affects CCAR1 abundance, we went on to assess whether this depends on a transcriptional or post-transcriptional regulation.

**Figure 3:**
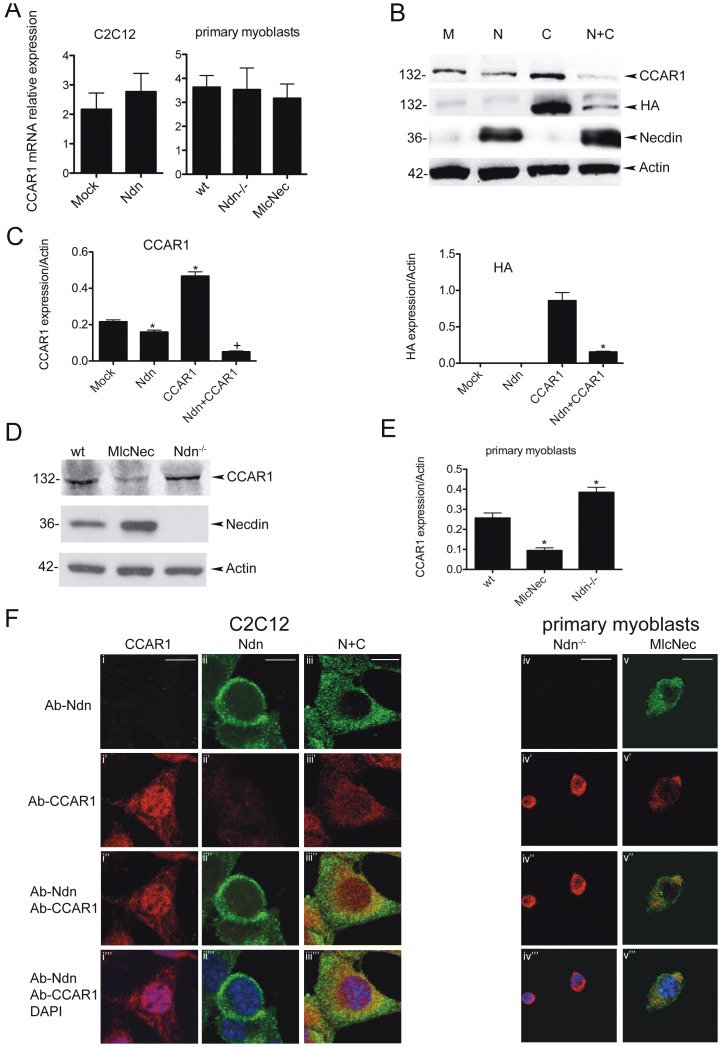
Necdin controls CCAR1 protein abundance in myogenic cells. A) CCAR1 mRNA relative quantification by qPCR using specific primers in C2C12 cells untransfected (Mock) or transfected with pCMV-FLAG-Necdin (Ndn) and in primary myoblasts from wt, Ndn^−/−^ and tgMlcNec (MlcNec) mice (data representative of respectively four and three independent experiments). β-Actin was used as internal standards. B) Representative western blot showing CCAR1 and necdin (β-actin as loading control) expression in C2C12 mock transfected (M) or transfected with pSG5-HA-CCAR1 (C) and/or pCMV-FLAG-Necdin (N or N+C). Both the endogenous and transfected CCAR1 were detected with the polyclonal anti-CCAR1, the transfected CCAR1 with anti-HA. C) Graphs show mean values ± s.e.m. obtained from the ratio of densitometric values of protein/β-actin bands on the blots in (B). Data are representative of three independent experiments. (* *p*<0,001 vs mock transfected; + *p*<0,005 vs CCAR1 transfected). D) Representative western blot showing expression of CCAR1 and necdin in primary myoblasts isolated from wt, Ndn^−/−^, tgMlcNec newborn mice. Proteins were detected using antibodies specific for CCAR1, necdin and β-actin as control. E) Graphs show mean values ± s.e.m. obtained from the ratio of densitometric values of protein/β-actin bands on the blot of the same experiments. Data are representative of three independent experiments. (* *p*<0,002 vs wt). F) Co-localization of necdin and CCAR1. Images taken at confocal laser scanning miscoscope (63x magnification, zoomed 1.67 times) showing co-immunostaining on C2C12 transfected with pSG5-HA-CCAR1 (i) and/or pCMV-Ndn (C2C12, CCAR1-Ndn-N+C) (ii, iii) and on primary myoblasts from Ndn^−/−^ (Ndn^−/−^) (iv) and tgMlcNec (MlcNec) (v) newborn mice. Panels i-ii-iii-iv-v show immunostaining using the specific monoclonal anti-Ndn (Ndn: Ab-Ndn-green); panels i'-ii'-iii'-iv'-v' show immunostaining using the polyclonal anti-CCAR1 (CCAR1: Ab-CCAR1-red). Co-immunostained images of anti-Ndn and anti-CCAR1 are shown in panels i''-ii''-iii''-iv''-v'' (Ab-CCAR1 + Ab-Ndn-yellow). Panels i'''-ii'''-iii'''-iv'''-v''' show merged images with nuclei stained with DAPI (merge Ab-CCAR1 + ab-Ndn + DAPI). Scale bars (i–iii) 41,75 µm; (iv–v) 44,7 µm.

We couldn't observe any changes in CCAR1 mRNA expression in neither wt primary myoblasts nor in C2C12 cells (Fig. 3A), while western blot analysis, using either the CCAR1 specific antibody or an HA specific antibody shows that in C2C12 untransfected or transfected with pSG5-HA-CCAR1 co-expression of necdin using the pCMV-FLAG-Necdin leads to a decreased expression not only of the endogenous CCAR1 protein (as shown before in Fig. 2C, D) but also of the transfected CCAR1 that carries a HA tag, suggesting that the effect of necdin is post-transcriptional and does not depend on signals on the endogenous CCAR1 gene (Fig. 3B, C).

We confirmed this result on primary myoblasts isolated from wt, tgMlcNec mice (necdin gain of function) and Ndn^−/−^ mice (necdin loss of function) [12]. Ndn^−/−^ cells show an increased level of CCAR1 respect to wt cells, while tgMlcNec mice overexpressing necdin specifically in skeletal muscle show a level of expression that is almost half of that of wt cells (Fig. 3D, E). Also in these samples, RNA levels were unchanged (Fig. 3A).

We also performed immunofluorescence experiments, on both C2C12 transfected with the different constructs and on primary myoblasts from Ndn^−/−^ and tgMlcNec newborn mice (Fig. 3F, Fig. S1). Interestingly we can observe that when necdin is not expressed or expressed at low levels (C2C12 transfected only with CCAR1 or Ndn^−/−^ primary myoblasts) CCAR1 localization is more abundant in the nucleus (Fig. 3F i, iv; Fig. S1 i, iv). On the contrary necdin overexpression not only reduces the level of CCAR1 but affects its localization that is now more cytoplasmic (C2C12 transfected with necdin and tgMlcNec primary myoblasts) (Fig. 3F ii, iii, v; Fig. S1 ii, iii, v). Of note, tgMlcNec primary myoblasts, even in proliferating conditions, show an enhanced differentiation ability as seen by the presence of elongated multinucleated myotubes (Fig. S1 v), respect to wt (Fig. 3D) and Ndn^−/−^ (Fig. S1 iv) as previously described [12].

These data confirm that necdin regulates CCAR1 protein abundance in myogenic cells.

MAGE proteins have been recently shown to interact with E3 RING proteins to promote protein ubiquitination, and consequently degradation via the proteasome [18], [23].

**Figure 4:**
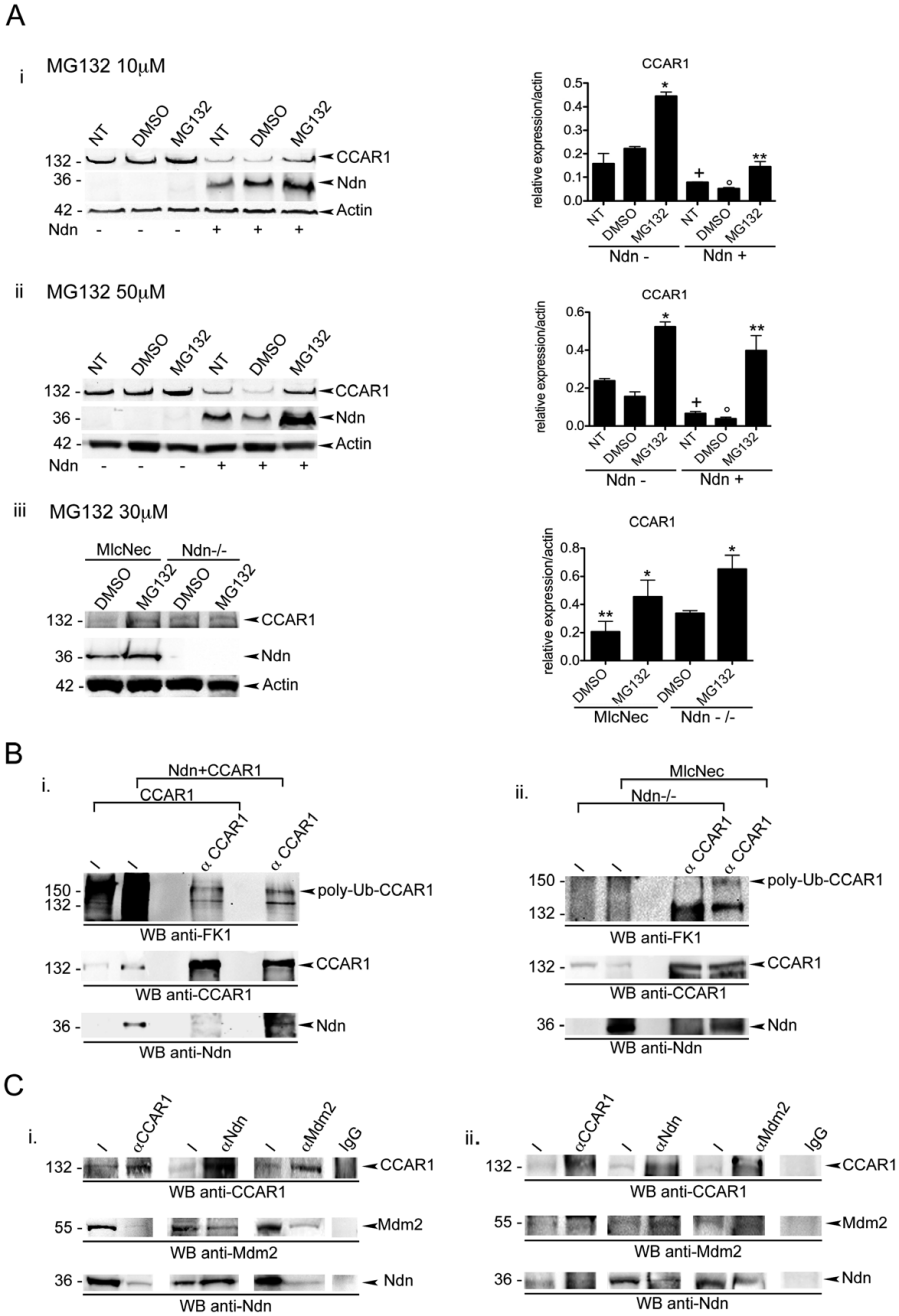
Necdin mediates CCAR1 degradation via the proteasome. A) i–ii Western blot on C2C12 cells not transfected (Ndn-) or transfected with pCMV-FLAG-Necdin (Ndn +) untreated (NT) or treated with two different concentrations of MG132 (MG132 i: 10 µM; ii: 50 µM) or an equal amount of DMSO as control (DMSO), using antibodies specific for CCAR1, Ndn and β-actin as loading control. Graphs on the right show mean values ± s.e.m. obtained from the ratio of densitometric values of protein/β-actin bands on the blot of the same experiments. Data are representative of three independent experiments. (* *p*<0,001 vs NT; ** *p<*0,001 vs NT transfected pCMV-Ndn, + *p*<0,001 vs NT untransfected, °*p<*0,002 vs untransfected treated with DMSO). iii. Western blot on primary myoblasts from Ndn^−/−^ (Ndn^−^/^−^) and from tgMlcNec (MlcNec) mice treated with the proteasome inhibitor MG132 (30 µM) or an equal amount of DMSO as control. Proteins were detected by western blot using antibodies specific for CCAR1, necdin or β-actin as control. Graph on the right shows mean values ± s.e.m. obtained from the ratio of densitometric values of protein/β-actin bands on the blot of the same experiments. Data are representative of two independent experiments. (* *p*<0,02 vs the relative DMSO treated sample; ** *p*<0,001 vs the DMSO treated Ndn^−/−^ cells). B) Analysis of the levels of ubiquitination of CCAR1 in absence and in presence of necdin. i. Proteins from C2C12 cells transfected only with pSG5-HA-CCAR1 (CCAR1) or transfected with pSG5-HA-CCAR1 and pCMV-FLAG-Necdin (Ndn + CCAR1) were immunoprecipitated using antibody anti-CCAR1 (αCCAR1) and analysed by western blot using antibodies specific for CCAR1, FK1 (recognizing mono and polyubiquitinated proteins) and necdin (Ndn). Input (I) represents 5% of the immunoprecipitated proteins. ii. Same co-immunoprecipitation experiment as in i. performed on proteins extracted from Tibialis anterior (TA) muscle from Ndn^−/−^ (Ndn^−/−^) and tgMlcNec (MlcNec) mice, immunoprecipitated samples were analysed by western blot using antibodies specific for CCAR1, FK1 (recognizing mono and polyubiquitinated proteins) and necdin (Ndn). Graphs of the densitometric values of ubiquitinated CCAR1 versus immunoprecipitated CCAR1 are shown in Figure S2. C) Interaction of MDM2, necdin and CCAR1. i. Co-Immunoprecipitation of MDM2, Ndn and CCAR1 in C2C12 cells transfected with pCMV-FLAG-Necdin, pCMV-Tag3b-Myc-MDM2 and pSG5-HA-CCAR1. Proteins were immunoprecipitated using polyclonal anti-CCAR1 (αCCAR1), monoclonal anti-MDM2 (αMDM2), polyclonal anti-necdin (αNdn) or unspecific IgG as control (IgG). MDM2, necdin and CCAR1 were detected in using respective specific antibody for MDM2, necdin (Ndn) or CCAR1. Input (I) represents 5% of the immunoprecipitated proteins. ii. Co-immunoprecipitation of MDM2, necdin (Ndn) and CCAR1 on TA of tgMlcNec mice. The experiment has been performed as in i.

To investigate whether protein degradation is involved in the regulation of CCAR1 protein expression, we first treated C2C12 myoblast cultures with the proteasome inhibitor, MG132. Western blot analysis showed that the treatment with 10–50 µM of MG132 for 6 h leads to an increased level of endogenous CCAR1 (Fig. 4Ai–ii). These results confirm that CCAR1 is degraded via the proteasome machinery.

When we administered MG132 to C2C12 overexpressing necdin, the effect of necdin on CCAR1 downregulation was abolished, indicating that necdin favours CCAR1 protein proteasomal degradation (Fig. 4Ai–ii).

This result was confirmed in primary myoblasts from Ndn^−/−^ and tgMlcNec newborn mice. The levels of CCAR1 in tgMlcNec, lower respect to Ndn^−/−^, are increased upon MG132 treatment.

To investigate how the proteasomal degradation of CCAR1 was dependent on necdin, we measured the level of CCAR1 ubiquitination. C2C12 were transfected with CCAR1 expressing vector alone or together with the necdin expressing vector. We immunoprecipitated CCAR1 using the CCAR1 specific antibody and we analysed the level of ubiquitinated proteins in the immunoprecipitated sample (Fig. 4B). Interestingly, necdin overexpression leads to an increased level of ubiquitin immunoprecipitated with CCAR1, even if CCAR1 is less abundant (Fig. 4Bi, Fig. S2i). Again we confirm this when we immunoprecipitated CCAR1 in protein extracts from the Tibialis anterior (TA) of Ndn^−/−^ and tgMlcNec 2 months old mice (Fig. 4Bii). We also observed that the band corresponding to the poly-ubiquitinated CCAR1 is increased in tgMlcNec respect to Ndn^−/−^ (Fig. 4Bii, Fig. S2ii).

These results indicate that necdin indeed favours the ubiquitination of CCAR1, thus leading to an increased degradation.

Finally we investigated whether necdin lead to CCAR1 degradation by mediating the action of specific E3 ligases. We decided to focus on one particular RING E3 ligases, MDM2. MDM2 is one of the ligase responsible for p53 ubiquitination [24], and we and others have described necdin regulating p53 expression, in particular in muscle cells [17], [25]. We therefore transfected C2C12 cells with vectors expressing FLAG-necdin, Myc-MDM2 and HA-CCAR1. Immunoprecipitation experiments using specific antibodies showed that MDM2 co-immunoprecipitates with both necdin and CCAR1 (Fig. 4D), and that CCAR1 and necdin binds to MDM2. We demonstrated that the three proteins bind to one-another by performing the same IP experiments on protein extracts from the Tibialis anterior (TA) of tgMlcNec 2 months old mice.

These data suggest that indeed necdin action on CCAR1 protein degradation is mediated by this E3 ubiquitin ligase.

## Discussion

A reciprocal balance between self-renewal and differentiation is the critical feature that maintains the stem cell pool and a failure in this process trigger an apoptotic fate decision, leading to a diminished number of stem cells. In the muscle, the mechanisms controlling satellite cell self-renewal and the choice of returning to quiescence after proliferation versus commitment and myogenic differentiation, remain incompletely understood, even if many candidate molecules have been found to play some role in different step of the process.

We have demonstrated that necdin is a critical player in sustaining survival of satellite cells and other myogenic stem cells such as the mesoangioblasts [12], [16]. Here we provide the first evidence of how necdin exerts its pro-survival activity in myoblasts. We show indeed that it physically interacts with a protein that plays an important role in regulating cell death, Cell Cycle and Apoptosis Regulatory protein-1 (CCAR1/CARP1) [19], [20], [22] and counteracts its pro-apoptotic effect. CCAR1 is a peri-nuclear protein that mediates apoptosis signalling by diverse agents, via activation of caspases 9 and 3, members of Jun N-terminal kinase (JNK) and p38 MAPK family of proteins. This protein was shown to interact with 14-3-3σ [19], [20], which in turn binds and negatively regulates MDM2 [26]. In absence of DNA damage this antagonizes MDM2-mediated p53 nuclear export and ubiquitination, which in turn destabilizes p53 [24], [27]. Upon cellular stress, DNA damage, or oncogenic activation, a decrease in MDM2 protein level and /or activity lead to the loss of p53-MDM2 interaction, p53 is stabilized and can act as a transcriptional activator of its target genes, promoting cell cycle arrest and apoptosis [24], [27]. CCAR1 has also been shown to directly function as a p53 co-activator in the transcriptional regulation of target genes, such as p21, in response to DNA damage stimuli [28]. Furthermore recent work in human cancer cell lines also demonstrates that CCAR1 is involved in Wnt/β-catenin pathway, co-operating with β-catenin and playing an important role in the activation of its target genes, therefore affecting cell growth and proliferation [29].

These data indicate a broad role for CCAR1 in both regulating cell cycle and apoptosis in the cell, exerting its functions by interacting with key cell growth and apoptosis transducers.

In skeletal myoblast, we found that CCAR1 expression is casually linked to an augmented sensitivity to cytotoxic stimuli and cell death. Necdin is able to counteract this effect by decreasing the levels of CCAR1. We show indeed an enhanced protein expression in myoblasts isolated from Ndn^−/−^ mice, while CCAR1 levels are decreased in myoblasts isolated from tgMlcNec mice, where necdin is overexpressed in a muscle specific way, or in C2C12 overexpressing necdin constitutively.

Necdin has been described to function as a transcriptional repressor, interacting with and suppressing functions of various proteins such as E2F1, p53, hnRNP U and NEFA [25], [30], [31], [32]. We have also described that necdin, in a model of muscle atrophy, counteracts the TNF-α pathway at different levels, including the modulation of the quantity of membrane bound TNFR receptor and of p53 [17]. Indeed in the case of CCAR1, necdin does not act as transcriptional repressor or activator, since CCAR1 mRNA levels are unchanged, but still leads to a reduced amount of CCAR1 protein.

This reduction of CCAR1 protein is dependent on the proteasome degradation pathway, indeed necdin overexpression increases ubiquitination of CCAR1 *in vitro* and *in vivo.* Blockade of the proteasome machinery abolish the action of necdin on CCAR1 degradation.

The MAGE family of protein, of which necdin is part, has been identified since many years, but their biochemical role, in particular the function of the MHD homology domain that characterizes them, has always been cryptic. Very recently a new light has been cast on this class of proteins, since it has been shown that they are able to interact, through the MHD domain, with specific RING proteins in particular RING containing E3 ubiquitin ligases, and in some cases even enhance their ubiquitin ligase activity [18]. This interaction promotes ubiquitination of target proteins, directing them to degradation via the proteasome machinery [18], [23], [33]. The MHD domain appears not to bind directly to the RING domain, but to other different motifs in the RING proteins, pointing to a flexibility of the MHD domain in terms of specificity. An action of necdin on protein stability, have been previously observed on p53 protein by the regulation of its acetylation level [34] and on HIF-1α, where again necdin overexpression lead to a decrease in HIF-1α protein in endothelial cells in both normoxic and hypoxic conditions [35]. CCAR1 on the other hand, has been found to interact directly or indirectly with different E3 ligases, such as APC/C, a component of APC proteome [36], involved in the degradation of cell cycle critical proteins.

Necdin may consequently facilitate the degradation of different proteins such as CCAR1 and p53, or possibly TNFR, by the directly interacting with specific members of the RING protein family. Until now the only E3 ligases shown to bind necdin, even if at low affinity, is Praja1 [37]. Here we found that necdin binds MDM2, and MDM2 immunoprecipitated samples contains both CCAR1 and necdin, thus suggesting that they are part of the same complex and that necdin may bridge MDM2 with its target protein CCAR1. Moreover in light of the fact that both CCAR1 and necdin interact with and regulates p53 activity [17], [28] and that p53 is a target of MDM2 [24], we could envisage a more complex network of interaction regulating cell survival in muscle cells. In depth studies are obviously required both to validate these interactions and functions, and more importantly to identify other E3 RING ubiquitin ligases to which necdin specifically binds to enhance the degradation of specific target proteins, and to get a full comprehension of the numerous functions of necdin in different cell types.

## Materials and Methods

The following reagents were used: polyclonal anti-necdin Ab (Upstate, NY, USA), monoclonal antibody anti-necdin mAb (Abnova, USA) polyclonal anti-CCAR1/CARP1 Ab (clone NB500-186, Novus Biologicals, USA), monoclonal anti-FLAG mAb, polyclonal anti-HA Ab and polyclonal anti-Actin Ab (Sigma, MO, USA), polyclonal anti-activated caspase-3 and, monoclonal anti-caspase 9 mAb (recognizing activated and non activated forms) (Cell Signalling, USA), monoclonal anti mono and poly ubiquitination, Ab clone FK-1 (Enzo Lifescience, USA), monoclonal anti-MDM2 (AbCam, USA). In immunoﬂuorescence analysis, primary antibodies were detected by appropriate Alexa-conjugated (Alexa 488 or Alexa 568) secondary antibodies (Cell Signalling, USA). In immunoblot analysis, primary antibodies were detected by chemiluminescence with appropriate horseradish peroxidase-conjugated secondary Abs, all purchased from Thermo Scientific (USA).

Two-Hybrid experiment in yeast was performed with BD Matchmaker Library Construction and screening Kit (BD Biosciences Clontech, USA).

For immunoprecipitation, Ez view anti-FLAG M2 affinity gel was supplied from Sigma (MO, USA) while Protein A and Dynal Immunoprecipitation Kit were from (Invitrogen, USA).

AnnexinV-FITC Apoptosis detection kit was provided by Immunostep (Spain).

Stausporine, MG132 and DAPI were from Sigma (MO, USA).

Cell culture media and sera were from Cambrex (Walkersville, MD, USA).

pSG5-HA-CCAR1 vector was kindly offered from Prof. Stallcup laboratory.

### Two-Hybrid experiment

GAL4 DNA-binding domain vector (pGBKT7) and GAL4 activation domain vector (pGAD-Rec) were purchased from CLONTECH. The cDNA library was generated from mRNA of myogenic precursors cells, by retrotranscription with specific LD primers as recommended by CLONTECH. These sequences were co-transformed along with pGBKT7 harbouring necdin full-length cDNA and pGAD-Rec linearized with Sma I into Saccharomyces cerevisiae AH109 cells. Transformants were grown first on leucine-tryptophan and histidine deficient synthetic dropout (SD) medium plates, and after the 2 mm colonies were grown on leucine, histidine, tryptophan and adenine deficient dropout medium supplemented with β-galactosidase activity as recommended by CLONTECH. Next, a specific LD primers PCR amplification and subsequent sequencing of possible interesting sequences was performed. The putative interactors were then confirmed by mating in yeast. Briefly, after isolation of pGAD-Rec-interesting cDNAs from AH109, were re-transformed alone in AH109 mat-a; while pGBKT7-Necdin, pGBKT7, or pGBKT7-Laminin (as negative control) were transformed in Y187 mat-α; the two strains were first grown in liquid YPD medium and then plated on SD/-leu, -His -Trp- Ade in triplicates.

### Plasmids cloning and cell culture

Full-length necdin cDNA was also cloned in pCMV-FLAG vector. Full-length MDM2 cDNA was cloned in pCMV-Tag3b-Myc vector.

C2C12 cells were from ATTC. Cells were cultured in DMEM, supplemented with 10% FBS, 100 U/ml Penicillin, 100 µg/ml Streptomycin, 10% Glutamine.

For transfections C2C12 cells were seeded 2.10^6^ on 100 mm dishes or 2,5.10^4^ on 6 well in DMEM 10% FBS and the following day cells were transfected with Lipofectamine LTX\Plus reagent (Invitrogen, CA, USA) using the specific plasmid vectors.

Primary myoblasts from C57/Bl6, Ndn^−/−^ and tgMlcNec newborn mice were isolated as described in [12] and plated at clonal density. Cells were grown in proliferation medium (DMEM supplemented with 20% FBS, 3% chick embryo, 100 U/ml penicillin, 100 μg/ml streptomycin, and 50 μg/ml gentamycin).

### Protein extracts and Immunoblot analysis

Cells were collected either in lysis buffer (Tris HCl pH = 7.4, 150 mM NaCl, 1 mM EDTA, 1% TRITON X-100, and protease inhibitor cocktail 1X (Sigma MO, USA) or in RIPA Buffer (50 mM TrisHCl pH = 7,4, 150 mM NaCl, 0,1% SDS, 0,5% Deoxycholate Sodium, 1% NP-40 and protease inhibitor cocktail 1X (Sigma MO, USA), cells were centrifuged 15 minutes at 13000 rpm at 4°C to discard cellular debris.

Muscle tissues from TA of C57/Bl6, Ndn^−/−^ and tgMlcNec 2 months old mice were dissected and homogenized in 100 mM NaHCO3, 1 mM EDTA, 2% sodium dodecyl sulfate, and protease inhibitor cocktail and centrifuged at 1,000 g for 10 min at 4°C to discard cellular debris. Sample preparation and Western blot analyses were performed as described in [38].

After electrophoresis, polypeptides were electrophoretically transferred to nitro-cellulose filters (Schleicher & Schuell, Dassel, Germany) and antigens revealed by the respective primary Abs and the appropriate secondary Abs, as already described (Deponti et al, 2007).

### Immunoﬂuorescence

Immunoﬂuorescence on the cell cultures (C2C12 myoblasts transfected with pSG5-HA-CCAR1 and/or pCMV-FLAG-Necdin, primary myoblasts isolated from C57/Bl6, Ndn^−/−^ and tgMlcNec newborn mice) was performed according to [39], using antibodies specific for necdin (mAb) and CCAR1 (pAb). For ﬂuorescent detection, we used appropriate secondary antibodies conjugated with either Alexa 488 or Alexa 568 (Cell Signalling).

### Real Time PCR

Real-time quantitative PCR (qPCR) was carried out with a real-time PCR system (Mx3000P; Stratagene). Each cDNA sample was amplified in triplicate by using the SYBR Green Supermix (Bio-Rad Laboratories) for β-actin (5′ GACCACCGCTCTTGTGTGT 3′ FW, 5′ GGATACCTCGCTTGCTCTGG 3′ REV), GAPDH (5′ TGAAGGTCGGAGTCAACGGATTTGGT 3′ FW, 5′ CATGTGGGCCATGAGGTCCACCAC 3′ REV), CCAR1 (5′GCAGCCACAGCCCTTATTAC 3′ FW, 5′ TTGATCCCCTCTGTCGTTTC 3′ REV).

### Co-immunoprecipitation

After, the pre-clearing with 2 ml wash buffer (Tris HCl pH = 7.4, 150 mM NaCl, 0.5% NP-40), 40 µl of Red Anti-FLAG M2 affinity gel (Sigma MO, USA) was incubated with 1,5 mg of fresh protein extracts in lysis buffer of C2C12 cells transfected with pCMV-FLAG and pSG5-HA-CCAR1 or pCMV-FLAG-Necdin and pSG5-HA-CCAR1. The resin while mixing was incubated overnight at 4°C then the supernatant was precipitated at 8000 g for 30 seconds and the beads were washed 10 minutes at 4°C for 3 times. After aspirating the final wash, pellets were re-suspended in 20 µl LAEMMLI buffer 4X, samples were vortexed and boiled before analysis by denaturating 10% SDS-Poliacrilammide gel electrophoresis. The membranes were incubated with antibodies against CCAR1, FLAG and necdin washed, and incubated with horseradish peroxidase-conjugated goat anti-rabbit and anti-mouse IgGs. Proteins were visualized by the chemiluminescence method (ECL western blot detection reagent, Thermo Scientific). For co-immunoprecipitation of CCAR1 and detection of necdin, protein A Agarose (Invitrogen) beads were used. 80 µl of beads were first incubated with 5 µg anti-CCAR1 Ab, anti-necdin ab or IgG as control for 1h at RT to crosslink antibody to the resin, meanwhile 1mg of protein extracts, from C2C12 transfected as above or from primary myoblasts cultured as described above, were incubated in 500 µl RIPA buffer for 1h at 4°C with 100 µl of protein A agarose beads to eliminate background. After centrifugation the pre-incubated protein extracts were transferred in protein A agarose + Ab beads o/n at 4°C. After three wash with wash buffer (EDTA 2 mM, 0,1%SDS,1% NP-40, 500 mM NaCl and protease inhibitor cocktail), the complexes were pelleted and resuspended with 20 µl of Laemmli Buffer 4X, the samples were boiled and vortexed, before analysis by 10% SDS-polyacrylamide gel eletrophoresis as above.

For immunoprecipitation of CCAR1 and detection of ubiquitinated proteins (in C2C12 cells transfected with pSG5-HA-CCAR1 and/or pCMV-FLAG-Necdin and in protein extract from TA extracted from Ndn^−/−^ or tgMlcNec 2 months old mice), 50 µl of magnetic dynabeads Protein A (Invitrogen) were first incubated with 5 µg Ab-anti-CCAR1 for 1 h at RT. The antigen containing sample (1,5 mg) was then added to the Dynabeads-Ab complex, incubated for 5 h at 4°C and finally eluted in NuPAGE LDS Sample Buffer/ NuPAGE Reducing Agent following the manufacturer's instruction. Anti-Ubiquitin (FK1 clone, for mono and poly-ubiquitination) was used for the detection of ubiquitinated CCAR1 in western blot.

This protocol was followed also to perform the immunoprecipitation of MDM2 and detection of CCAR1 and necdin, and viceversa, in C2C12 cells transfected with pCMV-FLAG-Necdin, pCMV-Tag3b-Myc-MDM2 and pSG5-HA-CCAR1 and TA of tgMlcNec mice (proteins extracted as described above).

### Cell death assay

C2C12 cells were transfected with pCMV-FLAG, pCMV-FLAG-Necdin, and /or pSG5-HA-CCAR1. After 60 h cells were treated with 200 nM Stausporine for 3 h (Caspase activation detection) or 12 h (Annexin V detection) and cells were collected.

For Annexin V detection, cells were stained with Annexin V-FITC and propidium iodide (PI) according to the kit's manufacturer's instructions and analyzed by flow cytometry (FACSCalibur; Becton Dickison) as described in [40]. Staining cells simultaneously with Annexin V-FITC and propidium iodide allows the discrimination of intact cells (Annexin V-FITC negative, PI negative), early apoptotic (Annexin V-FITC positive, PI negative) and late apoptotic or necrotic cells (Annexin V-FITC positive, PI positive).

For the detection of activated caspases, cells were lysed with RIPA buffer and analyzed for the expression of activated caspase-3 and -9 by western blot using specifc antibodies.

### Proteasome activity assay

C2C12 cells were transfected with pCMV-FLAG-Necdin as described above. Untransfected or transfected cells were treated for 6h with proteasome inhibitor, 10 µM or 50 µM MG132, and equal amount of DMSO, before being harvested and were then lysed with RIPA buffer. Primary myoblasts from Ndn^−/−^ or tgMlcNec mice, cultured as described above, were treated for 3 h with proteasome inhibitor, 30 µM MG132 and equal amount of DMSO, then harvested and were lysed with RIPA buffer.

The protein extracts were analysed by 10% SDS-polyacrylamide gel eletrophoresis and susbsequent western blotting with anti-CCAR1 antibody (Novus biologicals).

Anti-Ubiquitin (FK1 clone, for mono and poly-ubiquitination) was used for the detection of ubiquitinated CCAR1, only from protein extracts previously immunoprecipitated as described above.

### Image acquisition and manipulation

Fluorescent images were taken on confocal laser scanning microscopes (Leica TCS SP2 Laser Scanning Confocal; Plan Fluorlenses: 40× or 63.0×1.40 oil immerged, and enlarged images were zoomed 1,67 times from 63× acquisition; LSM 700 Confocal Laser Scanning Microscope, Carl Zeiss; Plan-Apochromat 63×/1.40 oil, Plan-Apochromat 40×/1.40 oil). The imaging medium was Oil; images were taken at room temperature. Images were assembled in panels using Photoshop 7.0 (Adobe). Images showing double or triple ﬂuorescence were separately acquired using the appropriate filters, and the different layers were merged with Photoshop 7.0.

Western blots and IP experiments were detected using a Kodak Image Station 440CF. Densitometric analysis of protein bands was performed using the ImageJ software. Images were assembled in panels using Adobe Photoshop 7.0.

### Statistical analysis

Data were analyzed with Microsoft Excel 12.2.3 and GraphPad Prism 5. The results are expressed as means ± SEM. Asterisks and crosses in the figure panels refer to statistical probabilities, measured in the various experimental conditions as detailed in the figure legends. Statistical probability values of <0.05 were considered significant.

### Ethical Statement

All animal work have been conducted according to the EEC and San Raffaele Ethical Committee guidelines under the licence IACUC n. 459, renewed on July 27th 2011 and valid to July 27th 2014. The San Raffaele Ethical Committee specifically approved this study.

## Supporting Information

Figure S1 Co-localization of necdin and CCAR1. Images taken at confocal laser scanning microscope showing co-immunostaining of necdin and CCAR1 in C2C12 transfected with pSG5-HA-CCAR1 (i) and/or pCMV-Ndn (ii, iii) (C2C12, CCAR1-Ndn-N +C) and in primary myoblasts from Ndn^−/−^ (Ndn^−/−^) (iv) and tgMlcNec (MlcNec) (v) newborn mice. Panels i-ii-iii-iv-v show immunostaining with the specific monoclonal anti-Ndn (Ndn Ab-Ndn-green); panels i'-ii'-iii'-iv'-v'show immunostaining with the polyclonal anti-CCAR1 (CCAR1: Ab-CCAR1-red); panels i''-ii''-iii''-iv''-v'' show co-immunostained images of anti-Ndn and anti-CCAR1 (Ab-CCAR1 + Ab-Ndn-yellow). Merged images in panels : i'''-ii'''-iii'''-iv'''-v''' highlight nuclei stained with DAPI (merge Ab-CCAR1 + Ab-Ndn + DAPI). Scale bars (i–iii) 25 µm; (iv–v) 17 µm.
(TIF)

Figure S2 i–ii. Graphs show mean values ± s.e.m. of the densitometric values of ubiquitin-CCAR1 bands in the CCAR1 immunoprecipitated samples in presence or absence of necdin, of the blots in Fig. 4Bi-ii. Data are representative of three independent experiments (i: refers to Co-IP in the C2C12 transfected cells experiment, Fig. 4Bi: * *p*<0,001 vs only CCAR1 transfected cells; ii: refers to Co-IP in TA extract, Fig. 4Bii: * *p*<0,002 vs Ndn^−/−^ TA muscle).
(TIF)
