# Supplementary figures and images for: Necdin Enhances Myoblasts Survival by Facilitating the Degradation of the Mediator of Apoptosis CCAR1/CARP1
Source: PLoS One. 2012 Aug 14;7(8):e43335. doi: 10.1371/journal.pone.0043335 (PMC3419192; doi:10.1371/journal.pone.0043335)

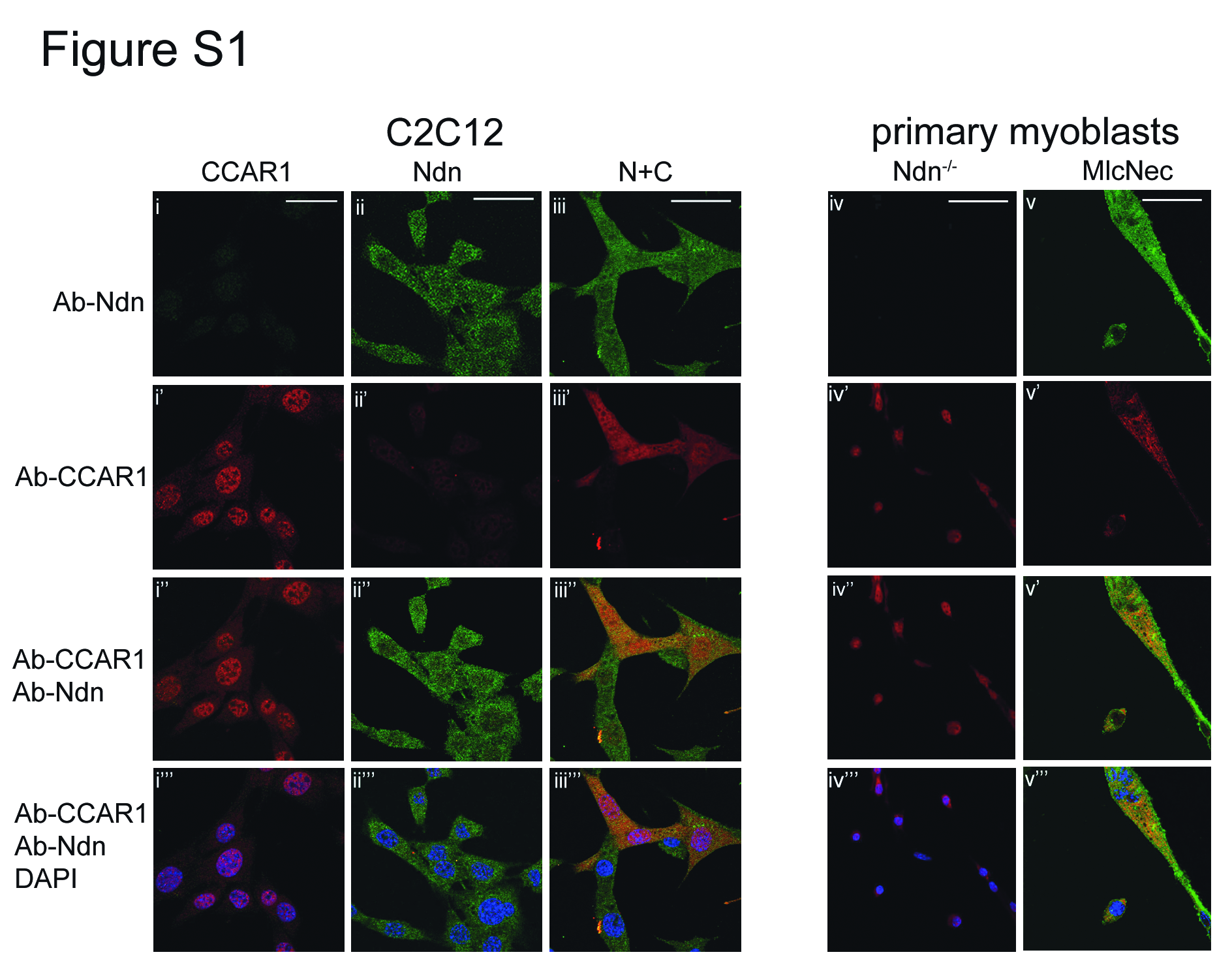

Supplement: Figure S1 — Co-localization of necdin and CCAR1. Images taken at confocal laser scanning microscope showing co-immunostaining of necdin and CCAR1 in C2C12 transfected with pSG5-HA-CCAR1 (i) and/or pCMV-Ndn (ii, iii) (C2C12, CCAR1-Ndn-N +C) and in primary myoblasts from Ndn−/− (Ndn−/−) (iv) and tgMlcNec (MlcNec) (v) newborn mice. Panels i-ii-iii-iv-v show immunostaining with the specific monoclonal anti-Ndn (Ndn Ab-Ndn-green); panels i'-ii'-iii'-iv'-v'show immunostaining with the polyclonal anti-CCAR1 (CCAR1: Ab-CCAR1-red); panels i''-ii''-iii''-iv''-v'' show co-immunostained images of anti-Ndn and anti-CCAR1 (Ab-CCAR1 + Ab-Ndn-yellow). Merged images in panels : i'''-ii'''-iii'''-iv'''-v''' highlight nuclei stained with DAPI (merge Ab-CCAR1 + Ab-Ndn + DAPI). Scale bars (i–iii) 25 µm; (iv–v) 17 µm. (TIF) [file pone.0043335.s001.tif]

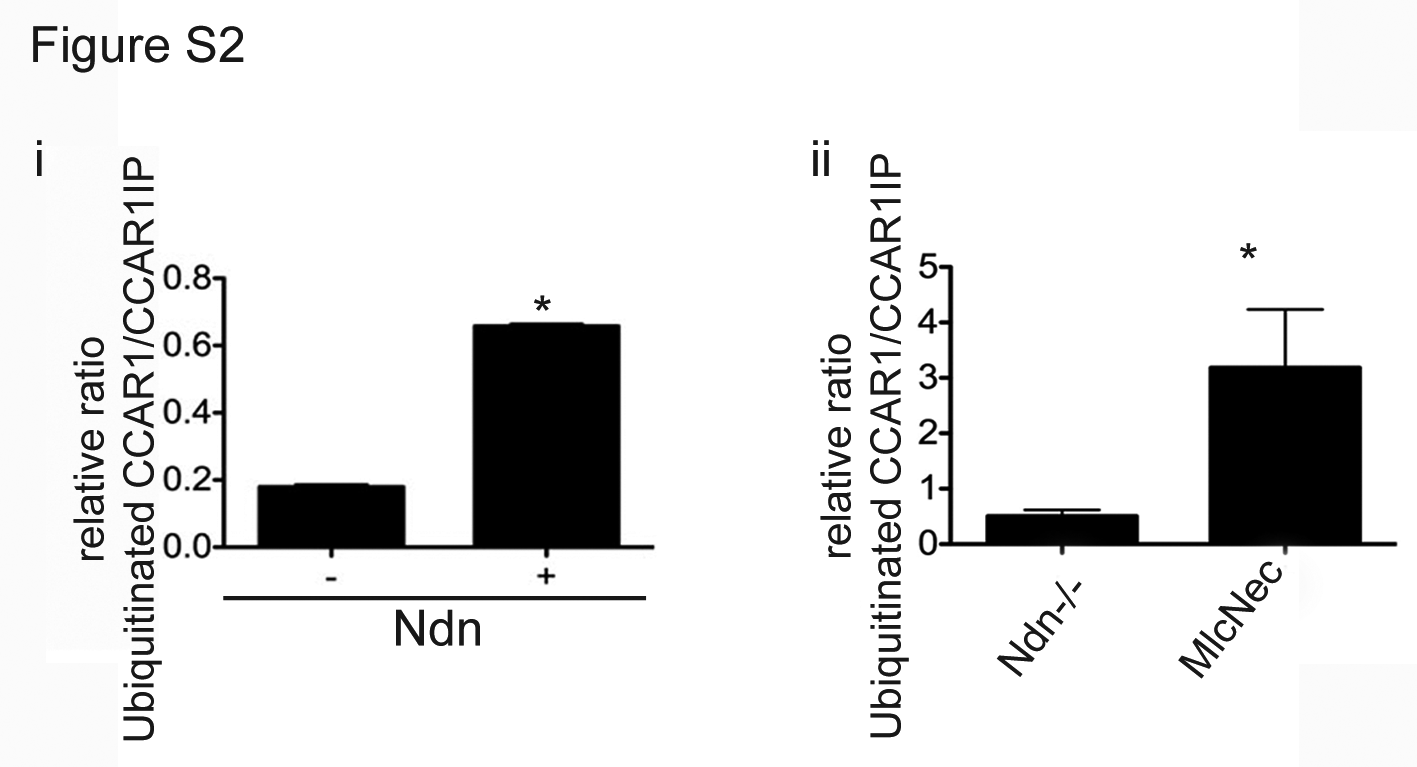

Supplement: Figure S2 — i–ii. Graphs show mean values ± s.e.m. of the densitometric values of ubiquitin-CCAR1 bands in the CCAR1 immunoprecipitated samples in presence or absence of necdin, of the blots in Fig. 4Bi-ii. Data are representative of three independent experiments (i: refers to Co-IP in the C2C12 transfected cells experiment, Fig. 4Bi: * p<0,001 vs only CCAR1 transfected cells; ii: refers to Co-IP in TA extract, Fig. 4Bii: * p<0,002 vs Ndn−/− TA muscle). (TIF) [file pone.0043335.s002.tif]
